# Effects of metformin on retinoblastoma growth *in vitro* and *in vivo*

**DOI:** 10.3892/ijo.2014.2650

**Published:** 2014-09-11

**Authors:** KATARZYNA BRODOWSKA, SOFIA THEODOROPOULOU, MELISSA MEYER ZU HÖRSTE, ELEFTHERIOS I. PASCHALIS, KIMIO TAKEUCHI, GORDON SCOTT, DAVID J. RAMSEY, ELIZABETH KIERNAN, MIEN HOANG, JOANNA CICHY, JOAN W. MILLER, EVANGELOS S. GRAGOUDAS, DEMETRIOS G. VAVVAS

**Affiliations:** 1 Retina Service, Angiogenesis Laboratory, Massachusetts Eye and Ear Infirmary, Department of Ophthalmology, Harvard Medical School, Boston, MA, USA; 2 Department of Immunology, Faculty of Biochemistry, Biophysics and Biotechnology, Jagiellonian University, Krakow, Poland

**Keywords:** toxicity, autophagy, proliferation, apoptosis, cyclin

## Abstract

Recent studies suggest that the anti-diabetic drug metformin may reduce the risk of cancer and have anti-proliferative effects for some but not all cancers. In this study, we examined the effects of metformin on human retinoblastoma cell proliferation *in vitro* and *in vivo*. Two different human retinoblastoma cell lines (Y79, WERI) were treated with metformin *in vitro* and xenografts of Y79 cells were established in nu/nu immune-deficient mice and used to assess the effects of pharmacological levels of metformin *in vivo*. Metformin inhibited proliferation of the retinoblastoma cells *in vitro*. Similar to other studies, high concentrations of metformin (mM) blocked the cell cycle in G0–G1, indicated by a strong decrease of G1 cyclins, especially cyclin D, cyclin-dependent kinases (4 and 6), and flow cytometry assessment of the cell cycle. This was associated with activation of AMPK, inhibition of the mTOR pathways and autophagy marker LC3B. However, metformin failed to suppress growth of xenografted tumors of Y79 human retinoblastoma cells in nu/nu mice, even when treated with a maximally tolerated dose level achieved in human patients. In conclusion, suprapharmacological levels (mM) of metformin, well above those tolerated *in vivo*, inhibited the proliferation of retinoblastoma cells *in vitro*. However, physiological levels of metformin, such as seen in the clinical setting, did not affect the growth of retinoblastoma cells *in vitro* or *in vivo*. This suggests that the potential beneficial effects of metformin seen in epidemiological studies may be limited to specific tumor types or be related to indirect effects/mechanisms not observed under acute laboratory conditions.

## Introduction

Retinoblastoma is the most common primary malignant intra-ocular tumor in infants and children. In the United States, it affects 12 per million children aged 0–4 years, representing 6.1% of all childhood cancers under the age of 5 years (1). Slightly more than half of the patients have the sporadic or non-inherited form of the disease, which results from the spontaneous inactivation of the retinoblastoma gene (RB1). Despite progress in the treatment of retinoblastoma, significant problems remain unsolved and metastatic disease is all too often fatal (2).

Although several treatment modalities are available for retinoblastoma, including local control of small to intermediate size tumors with laser and/or cryotherapy sometimes in combination with radiation and/or chemotherapy, or enucleation with or without systemic chemotherapy to control metastatic disease, each of them has major drawbacks, especially in pediatric patients. For example, conventional external beam radiation, which is used to control large tumors, has many complications, including an increased appearance of secondary malignancies, such as osteosarcoma. This complication occurs more frequently in patients with the hereditary-form of retinoblastoma. The 30-year cumulative incidence of second malignancies is >35% for patients who received external beam therapy vs. 6% for those patients without radiation (3). Intra-arterial chemiotherapy is currently novel treatment option for retinoblastoma, however, variables that affect blood flow can greatly affect drug delivery and therapy success (4–6). Also retinal and choroidal vasculopathy may occur in 10 to 20% of patients (7,8). Studies show that direct intravitreal injection of melphalan may be effective in controlling active vitreous seeds, however, major concern is the potential for tumor dissemination (6,9–12). Systemic chemotherapy used as a first line treatment for intraocular retinoblastoma with subsequent consolidation with photocoagulation, cryotherapy or radiotherapy has a recurrence rate of 24% by 5 years (13). This increases to 50% for patients with vitreous seeds (14). Recent analyses by several research groups (15–18) show success for local control approaching 90–100% for group A–C, but in less than 50% for group D (new international classification). In addition, significant morbidity with the chemotherapy has been described previously (19). One of the drugs used for chemotherapy (etoposide) is thought to be associated with increased incidence of acute myeloblastic leukemia although the actual number of cases implicated so far has been low with just ~20 cases reported in the literature (20). For these reasons, there is a pressing need for alternative treatment modalities for retinoblastoma with better safety and efficacy profiles.

Metformin is a biguanide drug that is widely used for the treatment of type II diabetes (4,21–23). A significant body of preclinical studies have shown that metformin decreases cancer cell viability and tumor growth in xenograft models (6,11,24–28). However, other studies have shown that metformin *in vivo* may accelerate tumor growth. For example, BRAF-mutant melanoma cells that are resistant to metformin *in vitro* show accelerated growth *in vivo* when treated with metformin (29). Likewise, metformin/AMPK activation promoted an angiogenic phenotype in the ERα negative MDA-MB-435 breast cancer model (30).

Some of the effects of metformin have been linked to activation of AMP-activated protein kinase (AMPK) in muscle, adipose and liver tissue (22,31). AMPK is activated by cellular stress resulting in the restoration of energy levels through regulation of metabolism and growth (32–34). Insufficient AMPK activity allows uncontrolled cell growth despite the conditions of cellular stress (such as those occurring during tumorigenesis). Furthermore, metformin has been shown to inhibit the mTOR pathway and S6K1 phosphorylation implicated in protein synthesis (4,6). Of note, these effects have been observed only at millimolar doses of metformin and recent studies indicate that metformin may exert its action through AMPK-independent mechanisms (6,11,24,28,35–41).

Thus the effects of metformin on the proliferation of cancer cells appear to be cell type dependent and not fully elucidated. For this reason, we investigated the effects of metformin on human retinoblastoma cancer cell lines *in vitro* and *in vivo*.

## Materials and methods

### Reagents

Metformin, MTT (3-(4,5-dimethylthiazol- 2-yl)-2,5-diphenyltetrazolium bromide) and ribonuclease-A were purchased from Sigma-Aldrich (St. Louis, MO, USA). Propidium iodide, calcein and DAPI were purchased from Invitrogen (Carlsbad, CA, USA). The following primary antibodies were purchased from Cell Signaling Technology (Danvers, MA, USA): phospho-ACC (Ser79), phospho-AMPK (Thr172), phospho-S6 ribosomal protein (Ser235/236), phospho-4E-BP1 (Thr37/46), p21 Waf1/Cip1, p27Kip1, LC3B, phospho-p38 MAPK (Thr180/Tyr182), phospho-Akt (S473), phospho-p44/42 MAPK (Erk1/2), β-tubulin, GAPDH. The following antibodies were purchased from Epitomics (Burlingame, CA, USA) cyclin E1, E2, D1, D3, A2, CDK4 and CDK2. Anti-Ki67 was purchased from Dako (Carpinteria, CA, USA), anti-CD31 and anti-CD11b from BD Bioscience (Franklin Lakes, NJ, USA).

### Cell culture

The human retinoblastoma cells WERI and Y79 (ATCC, Manassas, VA, USA) were grown in RPMI-1640 medium (Invitrogen, Grand Island, NY, USA) supplemented with 15% fetal bovine serum (ATCC), penicillin and streptomycin (both at 100 μg/ml; Invitrogen), 2 mM L-glutamine (Invitrogen) and 10 mM HEPES (Invitrogen). Cells were incubated at 37°C in a humidified atmosphere of 95% air and 5% CO_2_ and split when the cells reached approximately 80% confluence.

### Trypan blue exclusion test, growth curve and doubling time

Retinoblastoma cells were seeded in 6-well plates at a concentration of 4.5×10^5^ cells per well. On days 3, 6 and 9 cell number and viability was determined by trypan blue (0.4%) dye exclusion and growth-inhibition curves were drawn. Experiments were performed in triplicate with 2 wells per condition.

### Measurement of cell viability by the MTT assay

Cell viability was assessed by 3-(4,5-dimethylthiazol-2-yl)-2,5-diphenyltetrazolium bromide (MTT) assay. MTT assay is used to measure the reduction of a tetrazolium compound by the cellular mitochondria, producing an optically active soluble formazan.

Cells were cultured in 48-well plates at density 60,000 cells per well in 300 μl growth medium. After 1 and 3 days of treatment with metformin, MTT (5 mg/ml in PBS) was added to each well at a 1/10 volume. Cells were incubated for 1 h at 37°C and resuspended in DMSO. The absorbance at 595 nm was measured using a microplate reader. Data are displayed as percentage of control.

### Flow cytometry assessment of cell viability

Live and dead cells were quantified using the fluorescent probes calcein AM and DAPI. Cells were cultured in 6-well plates at 500,000 cells per 2 ml growth medium and were treated with 5 mM metformin for 48 h. The calcein was added at final concentration 0.1 μM and DAPI at 3 μM. The samples were read on Becton Dickinson FACScan. Results were analyzed with Summit 4.3 software.

### Flow cytometry assessment of the cell cycle

Cellular DNA content was assessed by flow cytometry. Cells were seeded in 6-well plates at density 500,000 cells per 2 ml growth medium and were treated with 5 mM metformin for 48 h. After overnight fixation in 75% ethanol, cells were suspended in PBS with DNase-free RNase A at final concentration 0.3 mg/ml and propidium iodide at final concentration 1 mg/ml. DNA content assessed on Becton Dickinson LSRII flow cytometer. Results were analyzed with Modfit LF software.

### Protein extraction and western blot analysis

For *in vitro* experiments, cells were incubated for 48 h in the presence or absence of metformin at various concentrations (12 μM to 10 mM). For *in vivo* experiments, tumor pieces were cut. The samples lysed in M-PER Mammalian Protein Extraction Reagent (Thermo-Scientific, Pierce Protein Research Products) with protease (according to manufacturer’s suggestions; Roche Applied Science) and phosphatase inhibitor cocktails (dilution 1:50; Thermo-Scientific, Pierce Protein Research Products). Total amount of protein (10 μg) was loaded onto a 4–12% Bis-Tris Gel (NuPAGE; Invitrogen). The electrophoresis was done using NuPAGE MOPS Running Buffer (Invitrogen) and then samples were transferred onto a PVDF membrane (Millipore, Billerica, MA, USA). The membranes were blocked for 45 min at room temperature in 5% wt/vol BSA, 1× TBS 0.1% Tween-20. The primary antibodies were diluted in 5% wt/vol BSA 1× TBS, 0.1% Tween-20 1:1,000 for all except CCNE1, E2, D1, D3, A2, CDK4 and CDK2 which were used at concentrations 1:5,000. After overnight incubation at 4°C, the membranes were washed three times 1× TBS 0.1% Tween-20 and incubated for 45 min at room temperature with the horseradish peroxidase-labeled secondary anti-rabbit antibody at 1:50,000 (Jackson Immuno Research, West Grove, PA, USA). The immunoreactive bands were visualized with ECL exposured to Fuji RX film (Fujifilm, Tokyo, Japan). The results were quantified using ImageJ software.

### Animals

All animal experiments complied with guidelines established by the Association for Research in Vision and Ophthalmology for the use of animals in ophthalmic and vision research, and were approved by the Animal Care and Use Committee of the Massachusetts Eye and Ear Infirmary (Boston, MA, USA). Four to five-weeks-old BALB/c (nu/nu) female mice were purchased from Charles River Laboratories (MA) and maintained in a facility under specific pathogen-free conditions in a climate controlled room with a 12 h light/dark cycle.

### Xenograft tumor growth assay

Xenograft tumors were established bilaterally in nu/nu mice by means of a single subcutaneous injection in each flank consisting of 4 million Y79 retinoblastoma cells suspended in 0.3 ml of a 1:1 mixture of ice-cold matrigel basement membrane matrix (BD Bioscience, MA, USA) and RPMI-1640 medium. Once a tumor mass became visible (within the week from injection of the cells), mice were randomly assigned to receive either daily peritoneal injections of metformin (250 mg/kg) or normal saline for 31 days. Two independent experiments were performed with five mice assigned to each group. The dose was based on the LD50 of metformin (420 mg/kg), as well as on human therapeutic and maximum prescribed doses for human patients (2,000–2,500 mg/day) (6,11). The tumor volume was monitored by external measurement in two dimensions with calipers every week and determined according to the equation: volume (mm^3^) = 4/3 × phi × (length/2) × (width/2)^2^ (9). Mice were weighted once a week.

### Immunohistochemistry assay and pathological evaluation

Five tumors from each group were frozen, cut into 10 μm sections and analyzed for retinoblastoma cell proliferation, vessel area and macrophage infiltration. Cryosections were also used for immunohistochemistry, first being fixed in 4% paraformaldehyde, blocked with 5% goat serum, and permeabilized with 0.1% Triton X-100. The sections were incubated in a humid chamber with primary antibodies, including anti-Ki67 (1:100), anti-CD31 (1:100) and anti-CD11b (1:100). A fluorophore-conjugated secondary antibody (Molecular Probes, Carlsbad, CA, USA) was used to detect fluorescence using a confocal microscope (Leica Microsystems, Wetzler, Germany). Nuclei were stained with DAPI. Cryostat sections were examined at random fields at ×20 magnification and the percentage of fluorescent-positive cells/DAPI-positive cells in each field was measured. Tumor vessel area was calculated as the number of image pixels that stained positive for CD31 per high-power field.

### TUNEL assay in tissue sections

Frozen 10 μm sections were prepared from tumors as above and stained with TUNEL cell death detection kit (Roche Diagnostics Corp., Indianapolis, IN, USA) according to the manufacturer’s recommendations. Sections were counter stained with DAPI and examined under an epifluorescent microscope (Leica Microsystems, Wetzler, Germany). Cryostat sections were examined at random fields at ×20 magnification and the percentage of TUNEL-positive cells/DAPI-positive cells in each field was measured.

### Serum levels of metformin, insulin-like growth factor-1 (IGF-1) and insulin-like growth factor binding protein 3 (IGFBP-3)

Retro-orbital blood was collected 3 and 15 h after metformin injection for ELISA testing and metformin levels assessment from all mice after euthanization. The samples were mixed with 4 mM EDTA and left at 4°C for 2 h, then centrifuged for 15 min at 180 × g. Serum levels of IGF-1 and IGFBP-3 were measured using a Mouse/Rat IGF-I and IGFBP-3 ELISA kit (R&D Systems, Minneapolis, MN, USA). Metformin levels were assayed (3 and 15 h after i.p. metformin injection), using high-performance liquid chromatography (NMS Lab, Willow Grove, PA, USA).

### Statistical analysis

The data are expressed as mean ± standard error of the mean (SEM). Statistical significance was evaluated using the one-way ANOVA test with Dunnett’s modification for multiple means comparison or t-test for two means. ^*^p<0.05 was considered statistically significant. Two-tailed tests were used for all comparisons.

## Results

### Metformin inhibits the growth and increases doubling time of human retinoblastoma cells in vitro at mM, but not μM levels

**Figure 1:**
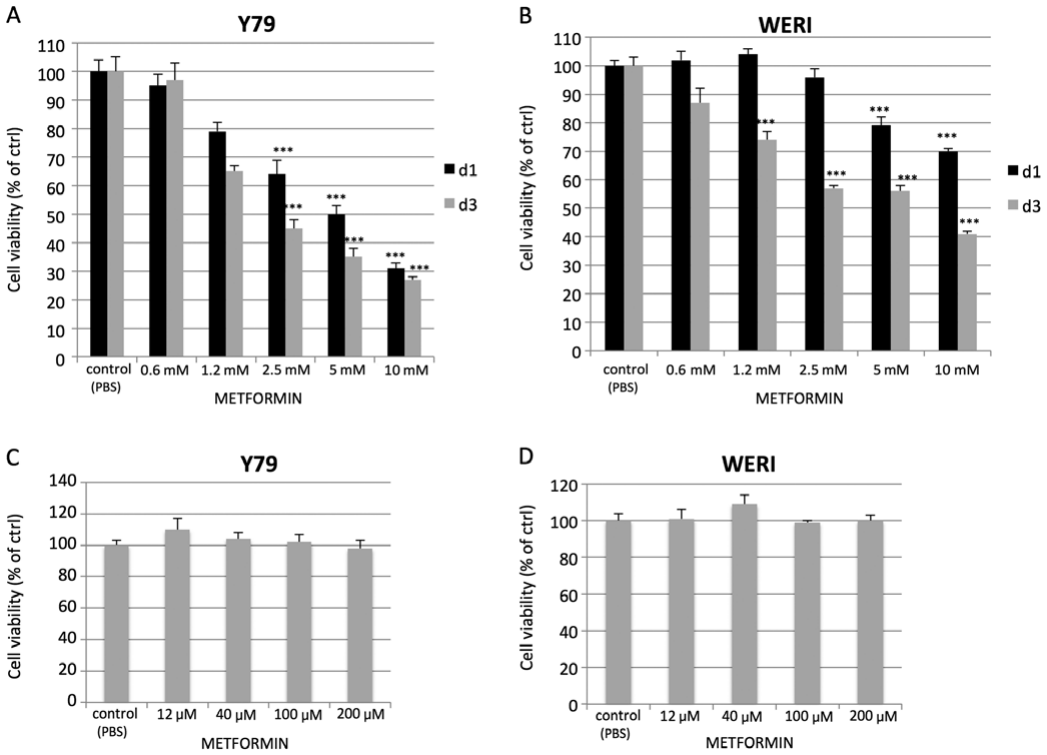
Metformin inhibits the proliferation of the retinoblastoma cells at mM, but not μM levels as measured by MTT. Retinoblastoma cell lines WERI and Y79 were treated with concentrations of metformin (12 μM to 10 mM) and cell viability was measured by MTT assay. (A–D) mM but not μM levels caused proliferation inhibition of both cell lines. The results are expressed as percentage of growth (%) relative to control values and are average of three independent experiments. Data are presented as mean ± SEM (n=12); ^**^p<0.01, ^***^p<0.005; d1, day 1; d3, day 3.

**Figure 2:**
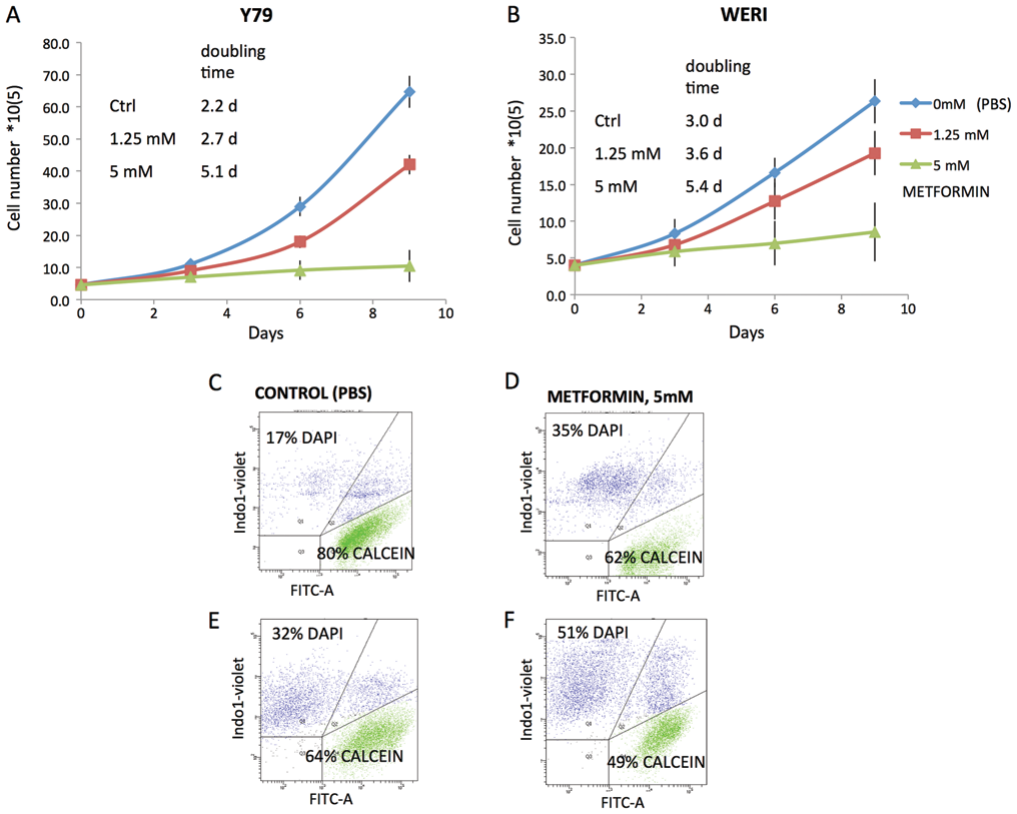
Metformin at higher mM levels increases the doubling time and causes cell death of retinoblastoma cells. (A and B) Retinoblastoma cell lines WERI and Y79 were treated with 1.25 and 5 mM of metformin for 48 h. Trypan blue exclusion test was performed on days 3, 6 and 9; metformin at mM levels caused proliferation inhibition; doubling time increased proportionally to metformin dose. The results are the average of three independent experiments. (C–F) The retinoblastoma cell lines Y79 and WERI were treated for 48 h with 5 mM of metformin, and cell viability and death was measured by calcein AM and DAPI staining using FACS; comparing to control, mM levels of metformin cause increased cell death and decreased viability (^**^p<0.01 for WERI and ^***^p<0.001 for Y79). The data are representative of three independent experiments (n=12).

In order to determine whether metformin affects human retinoblastoma cell viability and proliferation, we analyzed the effect of the drug on two human retinoblastoma cell lines: WERI and Y79. Cells were treated with various concentrations of metformin (12 μM up to 10 mM) and the viability was assed by the MTT assay. Increasing doses of metformin led to a corresponding reduction in cell viability but at doses in the mM range of concentrations (Fig. 1A and B). Reduced viability was not observed at μM concentrations (Fig. 1C and D). Assessment of cell growth and doubling time by trypan blue exclusion showed decreased growth rates in the presence of mM levels of metformin. Doubling time increased from 2.2 to 5.1 days for the Y79 cell line and from 3 to 5.4 days for the WERI cell line (Fig. 2A and B). Metformin treatment at 5 mM also increased the proportion of non-viable cells and decreased the proportion of viable cells (Fig. 2D and F) as judged by calcein AM/DAPI flow cytometry when compared to control (Fig. 2C and E).

### Metformin at higher mM levels leads to variable cell cycle changes in human retinoblastoma cells and to a global reduction in cell cycle regulators

**Figure 3:**
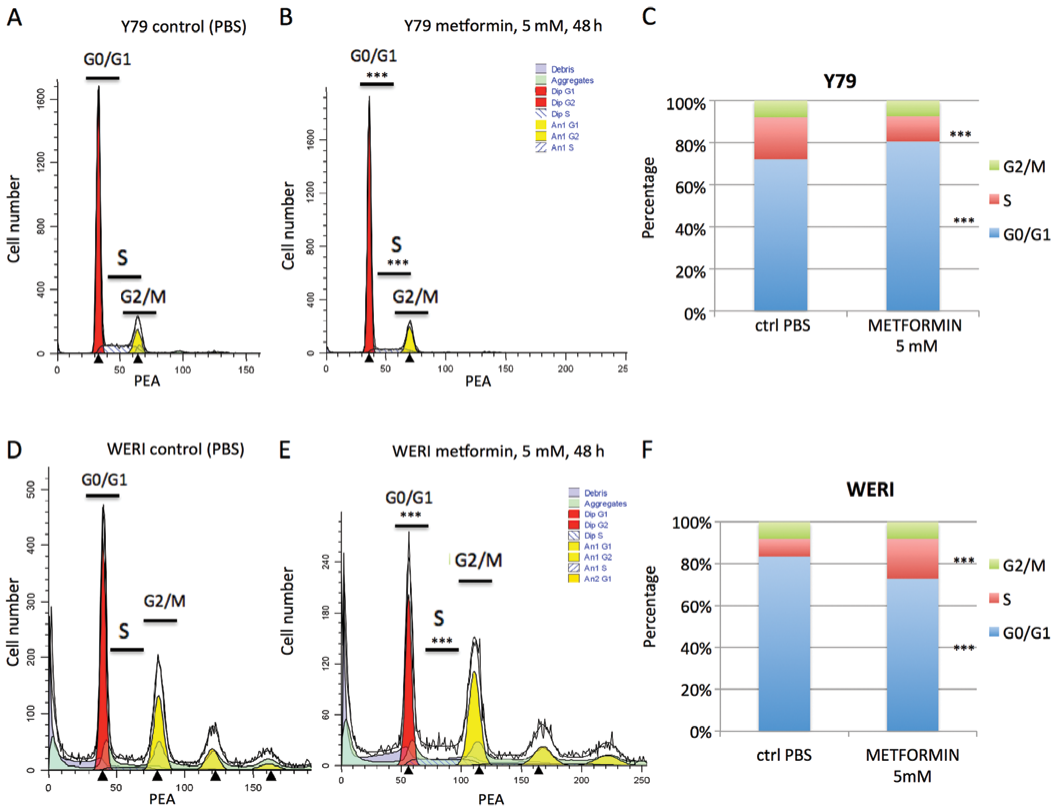
Metformin effects on the cell cycle in human retinoblastoma cells. Y79 retinoblastoma cells and WERI cells were treated with 5 mM metformin for 48 h. (A–C) Metformin caused cell cycle arrest in G0/G1 phase for Y79 while decreasing cell number in S phase. (D–F) Metformin caused cell cycle arrest in S phase for WERI cells while decreasing cell number in G0/G1 phase. All the data are graphically represented as percentage of cells in G0/ G1 phase, S phase, G2/M phase. The data are representative of three independent experiments (n=12).^***^p<0.001.

**Figure 4:**
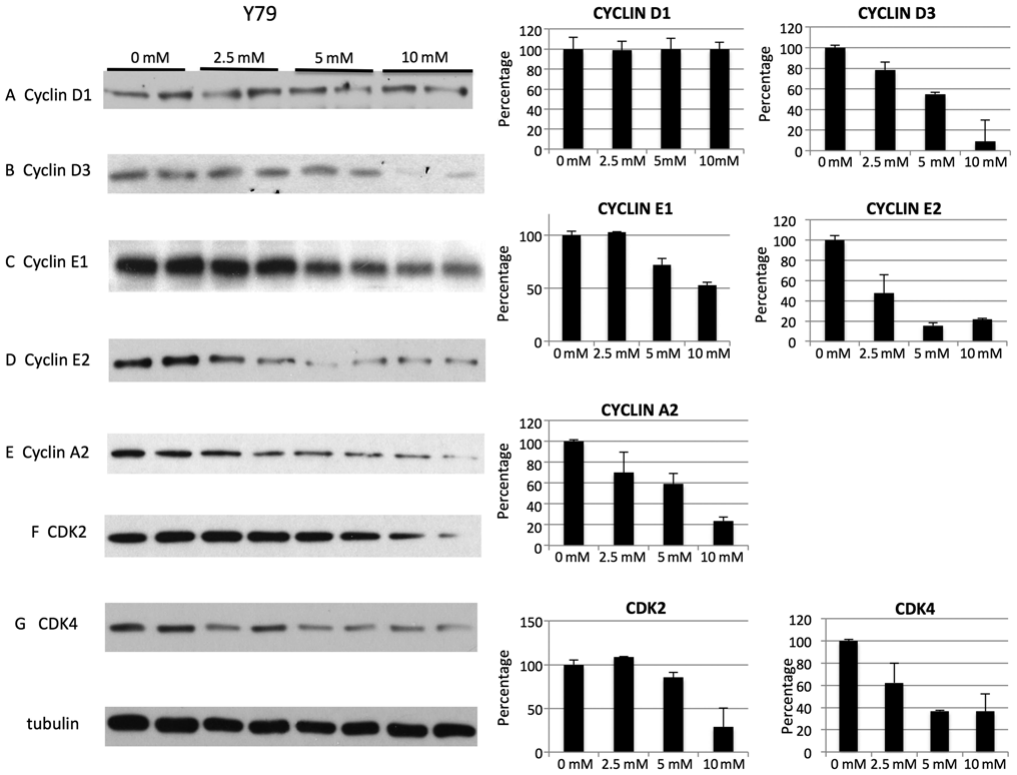
Metformin effect on cyclins D, E, A and Cdk2 and 4 in Y79 retinoblastoma cells. (A–G) Y79 cells were treated with 2.5, 5 and 10 mM metformin for 48 h and subjected to western blot analysis. Metformin caused downregulation of the cyclines D, E, A and CDK2 and 4 except cycline D1 for Y79. Data are representative of two independent experiments. Density values of the bands are graphically expressed relative to control. Data are shown as mean ± SEM (n=4).

**Figure 5:**
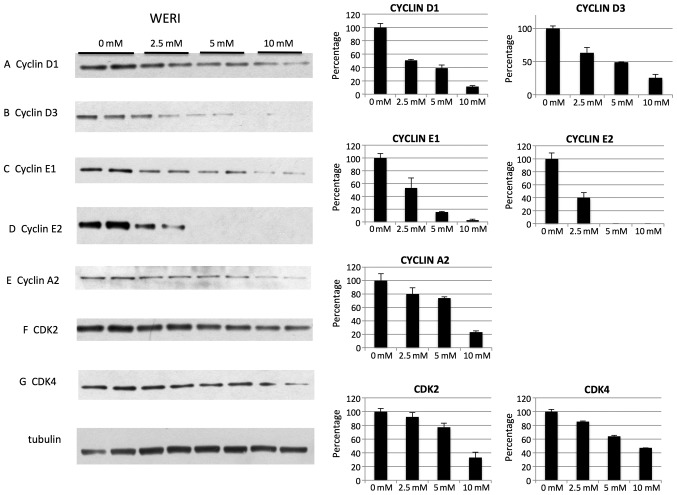
Metformin effects on cyclins D, E, A and Cdk2 and 4 in WERI retinoblastoma cells. (A–G) WERI cells were treated with 2.5, 5 and 10 mM metformin for 48 h and subjected to western blot analysis. Metformin caused downregulation of all cyclines D, E, A and CDK2 and 4. Data are representative of two independent experiments. Density values of the bands are graphically expressed relative to control. Data are shown as mean ± SEM (n=4).

**Figure 6:**
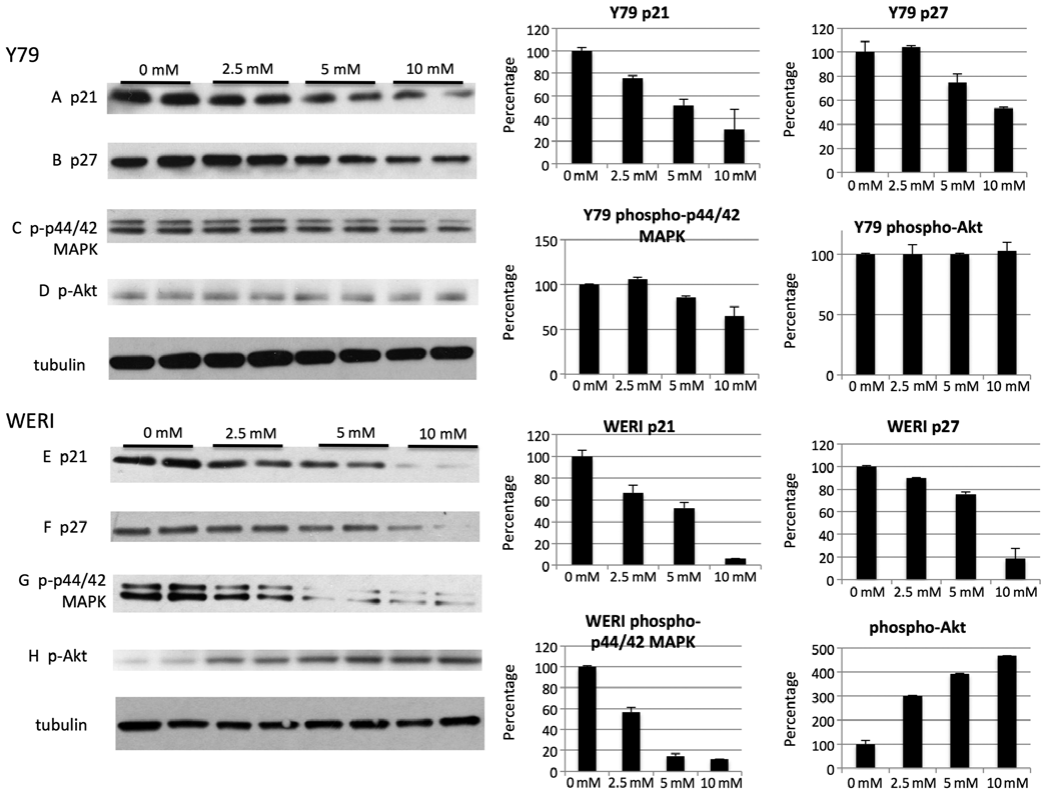
Metformin effects on the cell cycle regulators p21, p27 phospho-p44/42MAPK and phospho-Akt in Y79 and WERI cell lines. (A–D) Y79 cells were treated with 2.5, 5 and 10 mM metformin for 48 h and subjected to western blot analysis. Metformin caused downregulation of p21, p27, phospho-p44/42MAPK while it did not affect Akt. (E–H) WERI cells were treated with 2.5, 5 and 10 mM metformin for 48 h and subjected to western blot analysis. Metformin caused downregulation of p21, p27, phospho-p44/42MAPK while it upregulated Akt. Data are representative of two independent experiments. Density values of the bands are graphically expressed relative to control. Data are shown as mean ± SEM (n=4).

Previous reports have shown arrest in G0/G1 or S phase by mM levels of metformin (32). In our study, cell cycle analysis revealed that metformin treatment (5 mM for 48 h) of Y79 cells led to a statistically significant increase in cells in G0/G1 phase (72 to 81%, p<0.001), and a decrease in S phase (20 to 12%, p<0.001) (Fig. 3A–C). In contrast the reverse was seen when WERI were treated with metformin. There was a decrease in G0/G1 phase (83 to 73%, p<0.001) and an increase in cells in S phase (9 to 19%, p<0.001) (Fig. 3D–F). These cell cycle effects were not associated with specific cyclin and CDK changes but rather they were associated with non-specific global reduction in cyclins. For Y79 cell line on treatment with metformin we noted decrease of cyclin D3, E1, E2, A2 (Fig. 4B–E), cyclin dependent kinases CDK2 and CDK4 (Fig. 4F and G). Levels of cyclin D1 were not decreased for Y79 (Fig. 1A). For WERI cell line on treatment with metformin we noted decrease of cyclin D1, D3, E1, E2, A2 (Fig. 5A–E) as well as CDK2 and CDK4 (Fig. 5F and G). In addition, metformin treatment reduced CDK inhibitors p21 (Fig. 6A and E) and p27 (Fig. 6B and F) in both cell lines. Metformin reduced levels of positive cell growth regulators, such as phospho-p44/42 MAPK in Y79 (Fig. 6C) and WERI (Fig. 6G). Other cell proliferation and survival factors, such as phospho-Akt were unchanged in the Y79 cell line (Fig. 6D) but were found to be activated in the WERI cell line (Fig. 6H), suggesting that some of the effects of metformin on cell cycle may be non-specific.

### Metformin at higher mM levels inhibits the mTOR pathway, upregulates phospho-p38MAPK, autophagy marker LC3B and activates AMPK

**Figure 7:**
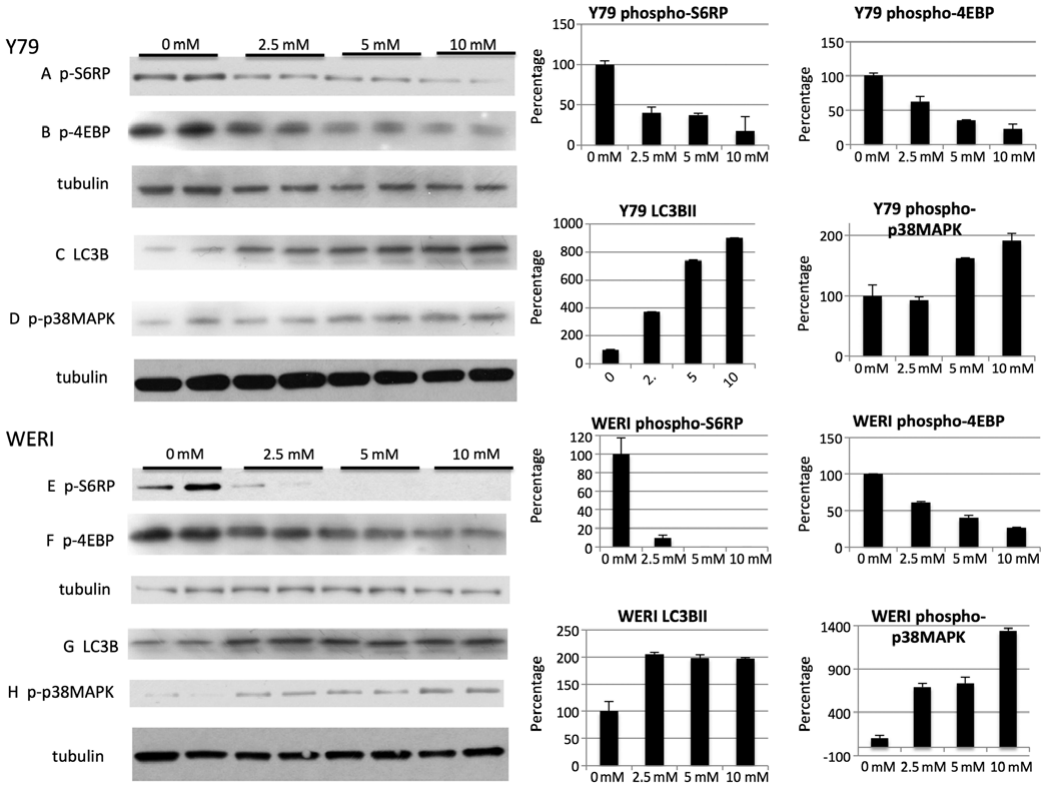
Metformin effects on phospho-p38MAPK, autophagy and the mTOR pathway in Y79 and WERI cell lines. (A–D) Y79 cells were treated with 2.5, 5 and 10 mM metformin for 48 h and subjected to western blot analysis. Metformin caused downregulation of p-S6RP, p-4EBP while it caused upregulation of LC3B I and II and upregulation of phospho-p38MAPK. (E–H) WERI cells were treated with 2.5, 5 and 10 mM metformin for 48 h and subjected to western blot analysis. Metformin caused downregulation of p-S6RP, p-4EBP while it caused upregulation of LC3B I and II and upregulation of phospho-p38MAPK. Data are representative of two independent experiments. Density values of the bands are graphically expressed relative to control. Data are shown as mean ± SEM (n=4).

**Figure 8:**
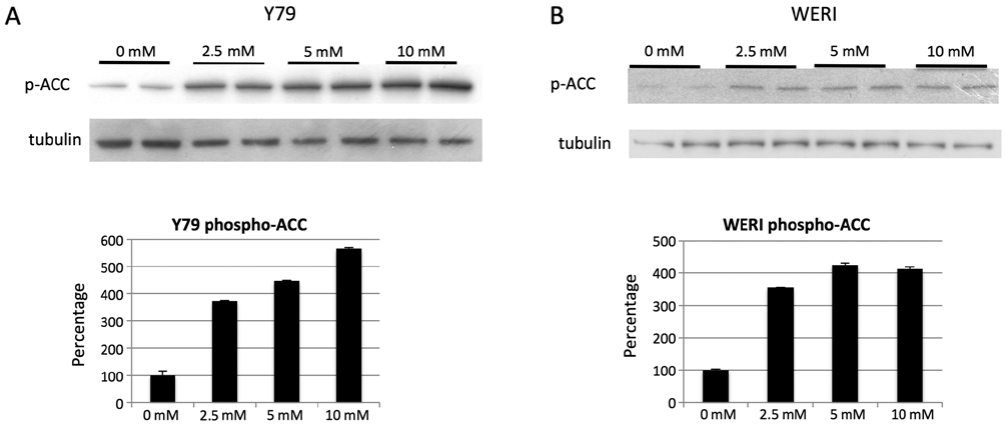
Metformin activates AMPK as judged by increase of phosphorylation of ACC in Y79 and WERI retinoblastoma cells *in vitro*. (A and B) Y79 and WERI retinoblastoma cells were treated with 2.5, 5 and 10 mM metformin for 48 h. Western blot analysis showed activation of phospho-ACC in a dose-dependent manner in treated cells compared to control cells. Data are representative of two independent experiments. Density values of the bands are graphically expressed relative to control. Data are presented as mean ± SEM (n=4).

Autophagy is usually activated under conditions of cell stress and is inhibited by the mTOR pathway, an intracellular signaling pathway important in apoptosis. Indeed mM levels of metformin decreased the mTOR pathway as judged by phosphorylation of S6RP (Fig. 7A and E) and 4E-BP1 (Fig. 7B and F) and led to variable increases in LC3B-I and LC3B-II protein levels (Fig. 7C and G). Similar to some (35,37,38) but not other studies (39,40) the induction of LC3 was associated with increases in p38 MAPK (Fig. 7D and H). Similar to other investigators (21) we found AMPK to be activated in retinoblastoma cells at the mM level as determined by phospho-ACC (Fig. 8A and B).

### Metformin at pharmacologic levels fails to suppress growth of human retinoblastoma xenografts in vivo

In order to evaluate the *in vivo* effect of metformin on retinoblastoma growth, heterotopic tumor xenografts of human Y79 retinoblastoma cells were established and mice were treated with metformin (250 mg/kg every 24 h) or equal volume of normal saline delivered i.p. (intraperitoneally). The dose of metformin was based on previous studies (11,28) and the LD50 for mice (420 mg/kg), as well as on the typical therapeutic and maximally prescribed human doses (2,000–2,500 mg/day) (6,11).

**Figure 9:**
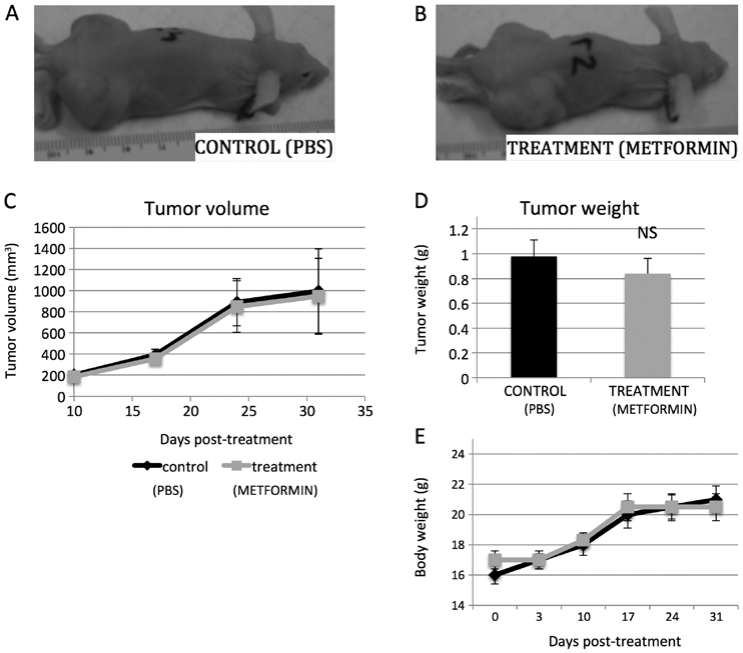
Metformin does not cause statistically significant inhibition of growth of xenografted tumors of Y79 human retinoblastoma cells in nu/nu immune-deficient mice. Human retinoblastoma Y79 cell heterotopic transplanted tumors were developed as described in Materials and methods. Mice were treated with metformin for 31 days. (A) Macroscopic appearance of the mice 31 days after transplantation of Y79 cells, without metformin treatment and (B) with 250 mg/kg/day treatment of metformin. (C) Tumor growth curves: mean volumes of PBS- vs. metformin-treated group on days indicated did not differ significantly. (D) Mean weights of tumors at autopsy of mice treated with PBS or metformin did not differ significantly. (E) Body weight of mice transplanted with Y79 cells with or without metformin treatment was not different.

In our *in vivo* experiments, metformin levels in mouse sera were on average 2.13 and 0.66 μg/ml for peak and trough, respectively (measured via high-performance liquid chromatography). The level of 2.13 μg/ml metformin equals about 12 μM. For comparison human peak levels are 1.03 (±0.33), 1.60 (±0.38), 2.01 (±0.42) for 500 mg p.o. (orally) daily, 850 mg p.o. daily or 850 mg p.o. taken three times per day, respectively (42). Despite achieving equivalent pharmacologic levels of metformin in mice, tumor growth was not significantly different than in the vehicle treated animals (Fig. 9A–C). The mean tumor weight, determined at necropsy, in the control mice was 0.98 g, as compared to 0.82 g in the metformin-treated mice (p=0.89, n=10, two independent experiments; Fig. 9D). The body weight of the tumor-injected mice was not found to differ significantly from controls (Fig. 9E).

**Figure 10:**
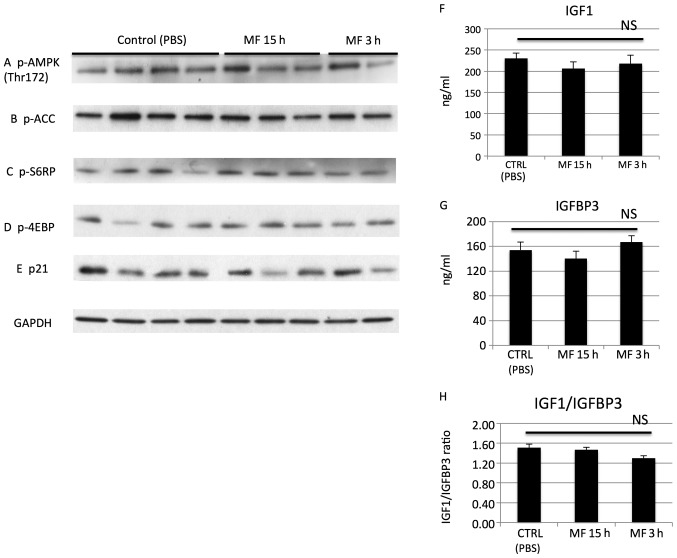
Effect of metformin on AMPK, mTOR and p21 *in vivo*. Tumors were collected 3 or 15 h after the last metformin injection for ELISA testing and proteins were extracted and western blot analysis was performed for indicated proteins. (A) Treatment of metformin did not show activation of AMPK, (B) increased phosphorylation of ACC, (C and D) inhibition of mTOR or (E) downregulation of p21 when compared to PBS treated mice. Data are representative of two independent experiments. Data are presented as mean ± SEM (n=4–8). (F and G) Serum levels of IGF1 and IGFBP3 of metformin treated mice failed to significantly differ when compared to PBS treated animals. (H) The ratio of IGF1/IGFBP3 did not differ when three groups were compared. Data are the mean ± SEM (n=5).

**Figure 11:**
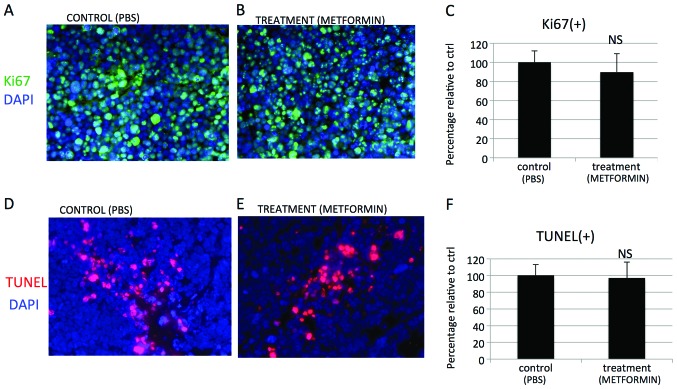
Metformin does not significantly alter proliferation or apoptosis of Y79-derived tumors xenografted into Balb/c nude mice. (A and B) Immunohistochemistry sections of representative tumors with Ki67 and (C and D) TUNEL staining. Nuclei were stained with DAPI (blue). (C–F) Quantitative analysis of Ki67(+)cells/DAPI(+) cells ratio and TUNEL(+)cells/DAPI(+) cells ratio in tumors was performed and results are expressed as a percentage of control. There was no difference between control and treated tumors when quantification was performed for (C) Ki67 and (F) TUNEL. Data are the mean ± SEM; Ki67 p=0.38 (n=5); TUNEL p=0.46 (n=5). Scale bars, 200 μm.

**Figure 12:**
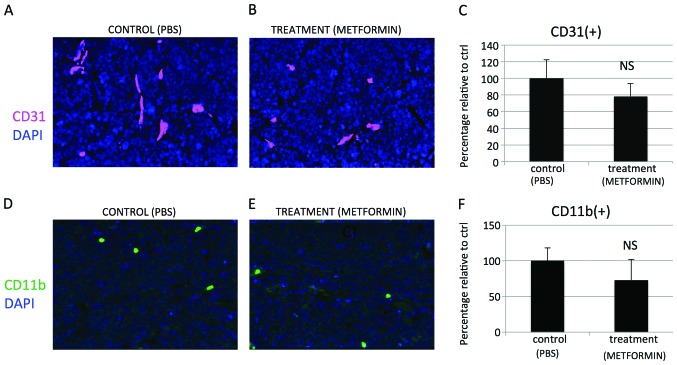
Metformin does not alter vascularity (CD31) or macrophage infiltration of xenografted tumors of Y79 human retinoblastoma in Balb/c nude mice. (A and B) Immunohistochemistry sections of representative tumors with CD31 and (D and E) CD11b nuclei stained with DAPI (blue). (C) Quantitative analysis of CD31(+)cells/DAPI(+) cell ratio and (D) CD11b(+)cells/DAPI(+) cell ratio in tumors was performed and results are expressed as a percentage of control. There was no statistically significant difference between control and treated tumors when quantification was performed for CD31 and CD11b. Data are presented as the mean ± SEM; CD31 p=0.14 (n = 5) and CD11b p=0.34 (n = 5); Scale bars, 200 μm.

We observed that metformin 3 and 15 h after i.p. administration did not affect proteins/pathways *in vivo* thought to be affected by metformin at mM levels *in vitro* such as AMPK, phospho-ACC, mTOR, p21 (Fig. 10A–E). Also the drug did not significantly affect the IGF1, IGFBP3 or the IGF1/IGFBP3 ratio in our experiments (Fig. 10F–H). When tumors were examined histologically significant changes of Ki-67 proliferative index [Ki67(+) cells/DAPI(+) cells; Fig. 11A–C] were not observed. Apoptosis labeling was similar in both groups [TUNEL(+) cells/DAPI(+) cells; Fig. 11D–F]. On treatment with metformin we observed a small, nonsignificant decrease in tumor vascularity (vessel area: μm^2^/hpf; Fig. 12A–C) and a small, nonsignificant decrease of infiltration by CD11b cells [CD11b(+) cells/DAPI(+) cells Fig. 12D–F].

## Discussion

Metformin, a drug used primarily for the treatment of type II diabetes, has been reported in epidemiological studies to reduce the incidence of certain cancers among diabetic patients (43). Initial studies examining the anti-proliferative effects of metformin have focused on tissues involved in insulin signaling and glucose/fatty acid metabolism, such as muscle and liver (4,21,23). However, the effects of metformin on other tissues or cells in culture have not been well characterized. Of the studies available (6,11,28), the anticancer effects of metformin are reportedly seen at mM levels, levels that are 100–1,000 times in excess of doses capable of being achieved by pharmacotherapy with metformin in humans. In addition, some conflicting data have arisen in *in vitro* and *in vivo* studies, with most indicating that the drug may have the potential to directly suppress tumor growth (11,24,28,32,44), while other reports indicate that metformin may not halt the growth of tumors (25,29,45,46). Increased tissue accumulation of metformin relative to blood levels have been hypothesized to explain metformin anticancer activity *in vivo*, although the concentration seen in most tissues still remains at the low 100 μM level (47). Other explanations proposed relate to metformin’s well-known effects on cholesterol, leptin, insulin levels and adiponectin, suggesting that some metabolic changes account for a reduction in tumor growth.

Some retrospective epidemiologic studies have revealed a decrease in the incidence of certain cancers in patients treated with metformin (48–50). The most recent meta-analysis suggests that metformin reduces the risk for colorectal cancer and hepatocellular cancer, but not for pancreatic, breast, gastric, prostate, bladder or lung cancer (50). Other case-control trials indicate that taking metformin is not associated with altered risk for esophagus cancer (51), endometrial cancer (52), lung cancer (53), colorectal cancer (54), prostate cancer recurrence and related mortality (55). Some trails suggest that metformin is associated with a decreased risk of pancreatic cancer but in women only (56). Others indicate that although metformin decreases risk of lung cancer, diabetics who develop lung cancer while receiving metformin may have a more aggressive cancer phenotype (57). Some trials show that only long-term use of metformin is associated with a tendency towards a decreased risk of ovarian cancer (58). Similarly long-term use of metformin (>5 years) but not short-term use was associated with lower risk for developing breast cancer compared with no use of metformin (59).

In this study, we examined the effects of metformin on human retinoblastoma growth *in vivo*, as well as *in vitro*, ranging from mM down to μM concentrations. We show that metformin inhibition of retinoblastoma cells *in vitro*, like all other cancer-related studies involving metformin, is seen at mM levels. Furthermore, levels similar to therapeutic levels achieved in humans (μM) do not have an impact on retinoblastoma growth either *in vitro* or *in vivo*.

High dose metformin has been shown to increase the activity of AMPK in various cell lines at mM levels similar to those used in our study (6,24,27,31,32,60) and activation of AMPK has been shown to be involved in cell proliferation (61,62). AMPK activation leads to inhibition of the mTOR pathway through tuberous sclerosis complex 2 (TSC2) (63) or directly without involvement of TSC2 after stimulation with pharmacological agent or with nutrient deprivation/stress (64). Indeed, in our study metformin activated the AMPK pathway in retinoblastoma cell lines at mM levels, as indicated by ACC phosphorylation and decreased phosphorylation of ribosomal protein S6 (a downstream effector of mTOR) and 4E-BP1 (a downstream effector of S6K). However, those effects were not observed *in vivo*. Energy deprivation and inhibition of the mTOR pathway (65) regulate autophagy (66), a process that maintains cellular homeostasis. Indeed, mM dose metformin inhibited the mTOR pathway associated with increased LC3-II expression (an autophagic marker). This is in agreement with some studies which showed that high dose metformin induces autophagy in cancer cells (25), however, others have not observed induction of autophagy (27).

Although use of metformin has been extensively used to study the AMPK pathway, like many pharmacological tools, it may have other unknown functions that are independent of its initially characterized action. Indeed, biguanides do not directly activate AMPK in cell free assays (67), and some studies have suggested that metformin mediates its effects completely independently of AMPK (24,36,68). Thus, to determine which proteins mediate the intracellular effect of metformin, further studies are warranted.

When the *in vitro* effects of metformin on the cell cycle are examined, it has been demonstrated that cells arrest either in the G1 phase (24,32,69), S phase (69), and/ or increase the proportion of cells in the sub-G0/G1 population (69) depending on the cell type. In our study, cell cycle analysis revealed that metformin treatment led to a significant increase of cells in G0/G1 phase and a decrease in S phase in Y79 cells, but the reverse was seen when WERI were treated with metformin (Fig. 3). Similarly to some researchers (24), we observed decrease of cyclin D1 at mM levels in WERI cell line, but in contrast to those reports, not in Y79 cells despite arrest in G0/G1 (Figs. 3A, 4A and 5A). Additionally, the different cell cycle changes observed in these two cell lines were not associated with any specific cyclin and CDK change, but a rather non-specific global reduction in cyclins (E1, E2, D3 and A2), cyclin-dependent kinase (CDK2 and CDK4) as well as the CDK inhibitors p27 and p21 (Figs. 4, 5 and 6). The downregulation of p27 at mM doses of metformin in our study is in contrast to research that showed upregulation of p27 in prostate cancer and ovarian caner cells (24,70), or no effect in breast cancer cells (69). Several studies have also indicated metformin may be involved in regulating the positive cell growth regulator phospho-Akt (69,71–73). In our study, the high (mM) dose of metformin *in vitro* resulted in variable effects on the two retinoblastoma cell lines. No effect was seen in the Y79 cell line, while in the WERI cell line metformin lead to increased phospho-Akt (Fig. 6D and H). The data on cell cycle, cyclins, and Akt taken together suggest that the *in vitro* high dose metformin effects on cell cycle of retinoblastoma cells may be non-specific.

Most *in vitro* experiments have shown effects in various cancer cell lines but they typically use concentrations in 2–50 mM, which are much higher than the plasma and tissue concentrations measured in individuals who receive recommended therapeutic doses (6,11,27,28). Studies with μM levels of metformin usually have little effect on cancer cell proliferation, as shown by our study and others (74,75). Yet several epidemiological studies have suggested that patients on metformin may have reduced cancer risk (76,77) and some animal studies have shown effects with μM levels (still almost 10-fold higher than the levels seen in patients on metformin) (78,79). In these studies (78,79) metformin was used with combination chemotherapy and was shown to have a preferential effect on tumor-forming, self-renewing cancer stem cells, which are resistant to mainstream chemotherapy, yet were found to be sensitive to metformin. Other additional hypothesis claim that metformin exerts its antitumor effects *in vivo* via its effects on insulin, IGF1 or IGFBP3 (reviewed in ref. 80), however, in our experiments the levels of IGF1, IGFBP3 or IGF1/IGFBP3 ratio remained unchanged (Fig. 10F–H).

Importantly, in our *in vivo* study, metformin administration lead to levels of the drug equivalent to those seen in patients on metformin, yet we did not detect statistically significant effect on tumor growth, apoptosis, proliferation, vascularity or infiltration by CD11b cells. It is possible that the effects of metformin may be cancer cell specific and/or may involve other pathways in the presence of concurrent chemotherapy. We can not exclude that long-term treatment with metformin may have cancer preventive effects for some cancer types which would be in agreement with some but not all clinical trials (4,21,23,42,43,58,59).

In conclusion, we found that while mM concentration of metformin inhibit growth of human retinoblastoma cell lines *in vitro*, μM levels comparable to those achieved *in vivo* do not. Furthermore, achieving therapeutic levels of metformin in plasma (μM levels) did not affect tumor growth in xenogratfs in Balb/c nude mice. Analysis of molecular signal changes suggests that the effects seen *in vitro* at mM metformin concentrations are possibly non-specific and due to the very high drug dose causing toxicity. Any potential beneficial effects of metformin seen in some, but not other, epidemiological studies of cancer require extensive further investigation with careful attention to the tumor type, as well as other indirect effects and mechanisms.
